# Confused Images Confused Eyes: A Case of Ultrasound Misdiagnosis of Pelvic Actinomycosis

**DOI:** 10.3390/diagnostics14171923

**Published:** 2024-08-31

**Authors:** Li Huang, Wen Xiong

**Affiliations:** Sichuan Academy of Medical Sciences & Sichuan Provincial People’s Hospital (SAMS & SPPH, Affiliated Hospital of UESTC), Chengdu 610014, China; 202222130337@std.uestc.edu.cn

**Keywords:** actinomycosis, imaging diagnosis, ovarian tumors, IUD

## Abstract

This article introduces a case of pelvic actinomycosis, which is easily confused with an ovarian malignant tumor. These images are from a 52-year-old woman who was admitted to hospital with difficulty defecating. Colonoscopy and biopsy indicated inflammatory changes within the intestinal tract, but the anti-inflammatory treatment was not effective. Later, she was readmitted due to abdominal pain and emaciation, and laboratory findings revealed mild anemia and inflammation. Various tumor markers are normal. CT suggested inflammatory lesions in the sigmoid colon and upper rectum. PET-CT considered a high metabolic mass originating from the mesentery. Ultrasound scan revealed a mixed-echo mass adjacent to the right side of the uterus, poorly demarcated from the rectum and right ovary, suggesting a neoplastic lesion. A biopsy of the right ovarian mass indicated suppurative inflammation, with negative antacid staining and microscopic observation of yellowish sulfur granules, suggestive of Actinomyces infection. Following a 12-month treatment regimen involving the removal of an intrauterine device and administration of penicillin, the patient’s condition markedly improved. Pelvic actinomycosis is usually characterized by abdominal pain accompanied by an abdominal mass, which is often related to an intrauterine device (IUD), and is very difficult to distinguish from pelvic tumors and tuberculosis, so it is necessary for doctors to understand its clinical and imaging features.

**Figure 1:**
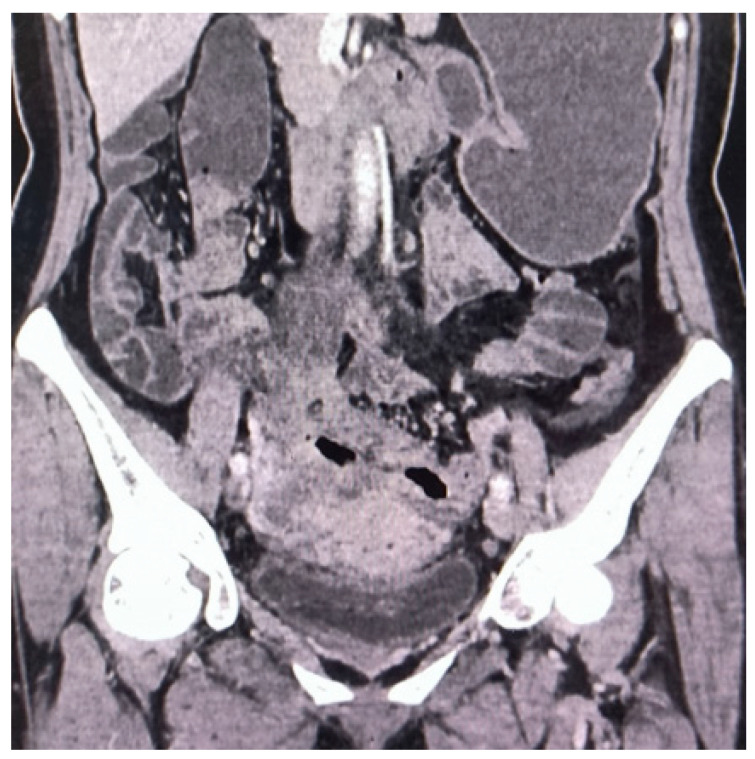
Contrast-enhanced computed tomography (CT) revealed roughening and thickening of the sigmoid colon and upper rectum walls, with luminal narrowing and uneven enhancement. In conjunction with laboratory examination (RBC 2.95 × 10^12^/L, Hb 80 g/L, WBC 12.3 × 10^9^/L, CRP 53.44 mg/L, CEA 1.73 ng/mL, AFP3.64 ng/mL, CA125 19.2 U/mL, CA19-9 8.83 U/mL), CT indicated possible inflammatory lesions with pelvic lymph node enlargement.

**Figure 2:**
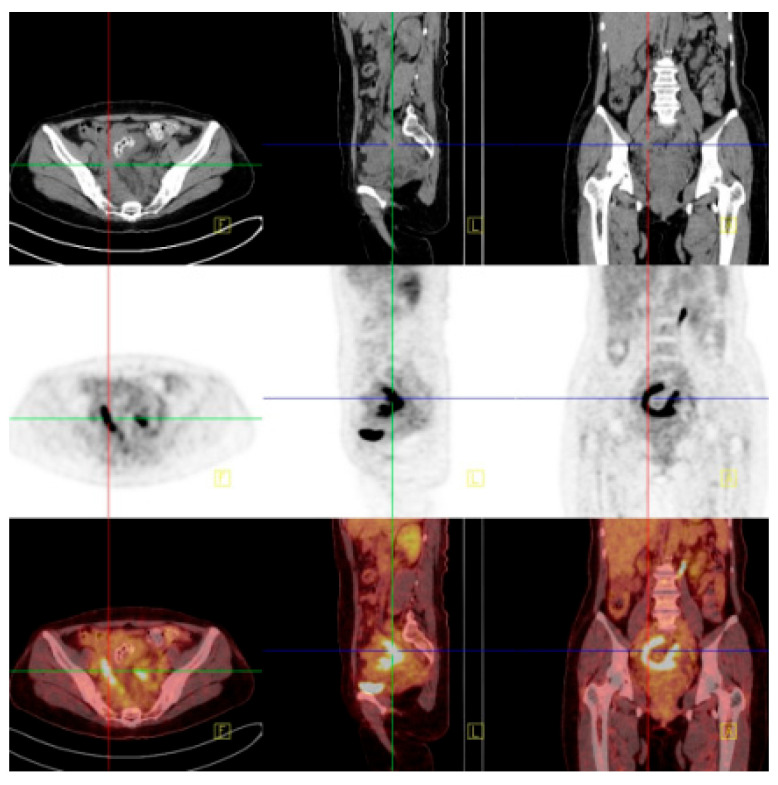
Positron emission tomography–computed tomography (PET-CT) reveals an irregular high-density mass in the pelvic mesentery, forming a ‘C’ shape enveloping the sigmoid colon from the right, with unclear demarcation from the sigmoid colon and markedly increased FDG uptake. FDG can be used as an important indicator to distinguish benign and malignant lesions by reflecting the level of glucose metabolism in the lesion, but it does not have absolute specificity. Tuberculosis and inflammatory lesions can also show increased FDG. There may be chronic granulation tissue hyperplasia near actinomycete abscesses or sinuses, and the infiltration of various inflammatory cells, tissue cells, and fibroblasts, resulting in high uptake of 18F-FDG.

**Figure 3:**
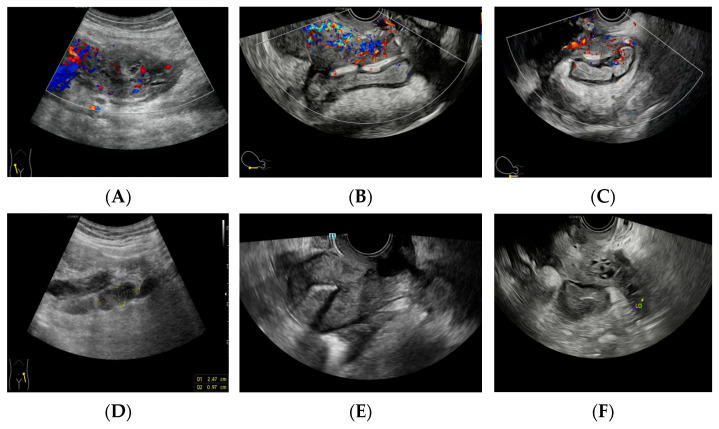
The preoperative ultrasound detection of pelvic mass: (**A**) ultrasound detection of a mass of about 8.8 × 4.4 × 4.8 cm in the right adnexa, with abundant blood flow signals, RI: 0.65. (**B**) The mass is not clearly demarcated from the rectum, sigmoid colon, and sacroiliac ligament. (**C**) Ultrasound shows thickening and decreased echo of the intestinal wall behind the uterus, with abundant blood flow. (**D**) Multiple enlarged lymph nodes in the bilateral iliac fossa. (**E**) A copper-bearing intrauterine contraceptive device, in the normal position. (**F**) The size and shape of the left ovary are acceptable.

**Figure 4:**
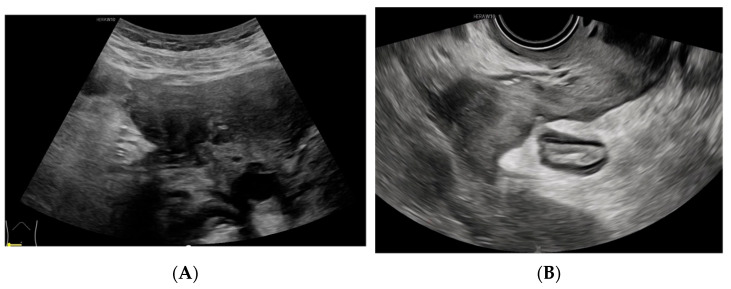
(**A**) A gradual reduction in the mass. (**B**) The eventual resolution of the mass, confirming a diagnosis of Actinomyces infection. Pelvic actinomycosis, a rare subacute to chronic granulomatous disease, constitutes approximately 3% of all actinomycosis cases [1]. Long-term IUD placement can lead to endometrial damage, facilitating the invasion of Actinomyces into the subendometrial layer. This invasion often extends to the fallopian tubes and ovaries, where Actinomyces secrete protein hydrolases that can exert a malignancy-like invasive effect and provoke an infection-like inflammatory response [2]. The clinical and imaging features of pelvic actinomycosis are not distinct, making its diagnosis challenging. Preoperative diagnosis is accurate in less than 10% of cases [3]. Although the imaging characteristics of pelvic actinomycosis are not unique, they also have some similar features with other diseases [2,4]. CT may reveal a thick-walled cystic or solid mass with heterogeneous enhancement. Ultrasound typically shows cystic–solid mixed echoes with abundant blood flow signals. In the early stages, the disease may present as an isolated mass. As the disease progresses, the mass enlarges, invading and compressing adjacent organs, and becomes increasingly irregular in shape and poorly demarcated from surrounding structures, complicating preoperative diagnosis. The affected intestinal wall may exhibit echogenic signs of inflammation due to invasion. The urinary tract can also demonstrate fluid dilatation due to compression and obstruction. On PET/CT, the mass often displays high fluorodeoxyglucose (FDG) uptake. Reflecting on this case, the localization of the lesion posed significant challenges. The copious inflammatory exudate and extensive adhesions within the pelvis obscured clear demarcation on tomographic images. Consequently, based on the patient’s prolonged gastrointestinal symptoms, both CT and PET-CT erroneously suggested an intestinal tract and mesentery origin. In contrast, ultrasonography, employing real-time scanning through various modalities and perspectives, accurately localized the lesion to the right adnexal region. Qualitatively, differentiating this disease from ovarian malignancy and pelvic tuberculosis is exceptionally challenging. Continuous differentiation throughout the course of the disease is imperative, especially considering the high failure rate (up to 50%) of Actinomyces cultures [1]. This necessitates extreme caution in postoperative pathological differentiation. Patients with ovarian cancer, predominantly older women, typically present with abdominal pain, distension, and cachexia, often lacking symptoms like chills and high fever. These cases are characterized by a marked elevation in CA125 levels. Unlike the extensive adhesions and sinus tract formation in the late stage of actinomycosis, larger ovarian tumors on imaging are often accompanied by significant abdominal or pelvic effusions and extensive lymphadenopathy. In this case, real-time dynamic ultrasound provided distinct advantages in determining the origin of the mass, assessing the extent of pelvic adhesions, and evaluating the involvement of surrounding tissues. It was also preferred for post-surgical efficacy assessment, playing a pivotal role throughout the diagnostic and treatment process.

## Data Availability

No new data were created or analyzed in this study. Data sharing is not applicable to this article.
